# Recent Advances in Molecular Diagnosis of *Pseudomonas* *aeruginosa* Infection by State-of-the-Art Genotyping Techniques

**DOI:** 10.3389/fmicb.2018.01104

**Published:** 2018-05-28

**Authors:** Jian-Woon Chen, Yin Yin Lau, Thiba Krishnan, Kok-Gan Chan, Chien-Yi Chang

**Affiliations:** ^1^International Genome Centre, Jiangsu University, Zhenjiang, China; ^2^Division of Genetics and Molecular Biology, Institute of Biological Sciences, Faculty of Science, University of Malaya, Kuala Lumpur, Malaysia; ^3^School of Chemistry and Biosciences, University of Bradford, Bradford, United Kingdom

**Keywords:** molecular diagnostics, *Pseudomonas aeruginosa*, polymerase chain reaction, pulse-field gel electrophoresis, next generation sequencing

## Abstract

*Pseudomonas aeruginosa* is a rod-shaped Gram-negative bacterium which is notably known as a pathogen in humans, animals, and plants. Infections caused by *P. aeruginosa* especially in hospitalized patients are often life-threatening and rapidly increasing worldwide throughout the years. Recently, multidrug-resistant *P. aeruginosa* has taken a toll on humans’ health due to the inefficiency of antimicrobial agents. Therefore, the rapid and advanced diagnostic techniques to accurately detect this bacterium particularly in clinical samples are indeed necessary to ensure timely and effective treatments and to prevent outbreaks. This review aims to discuss most recent of state-of-the-art molecular diagnostic techniques enabling fast and accurate detection and identification of *P. aeruginosa* based on well-developed genotyping techniques, e.g., polymerase chain reaction, pulse-field gel electrophoresis, and next generation sequencing. The advantages and limitations of each of the methods are also reviewed.

## Introduction

*Pseudomonas aeruginosa* is a common environmental microorganism which is widespread in nature. It is a ubiquitous Gram-negative bacterium which increasingly recognized as an emerging opportunistic pathogen of clinical relevance due to its high morbidity and mortality infection rate in healthcare settings especially among immunocompromised individuals and other highly vulnerable patients. *P. aeruginosa* has been classified as an ESKAPE (*Enterococcus faecium*, *Staphylococcus aureus*, *Klebsiella pneumoniae*, *Acinetobacter baumannii*, *P. aeruginosa,* and *Enterobacter* species) pathogen; one of the six highly antibiotic resistant bacteria (Rice, 2008). It is also listed as a critical priority pathogen “for which new antibiotics are urgently needed" by World Health Organization [WHO], 2017. The detection of *P. aeruginosa* at early aggressive antibiotic treatment is significant in order to prevent or to postpone chronic lung colonization for cystic fibrosis patients (Starner and McCray, 2005; Deschaght et al., 2011; Douraghi et al., 2014) and burn infections which could lead to consequences like pneumonia, sepsis, and necrosis (De Vos et al., 1997; Edwards-Jones et al., 2003; Mayhall, 2003). There is therefore a need for sensitive and specific *P. aeruginosa* detection tests in clinical laboratory for rapid triage and early aggressive targeted therapy.

Molecular diagnostics (MDx) has taken a prominent place and has shown advantages in the clinical diagnostic laboratory for routine detection, fingerprinting, and epidemiologic analysis of infectious microorganisms. Firstly, MDx minimizes the requirement for cultivation, which reduce the time required for morphology and biochemistry diagnosis. Disadvantages of cultivation include the selection of microbe-specific artificial media, the ability of the microbe to propagate on the selected media and long incubation period (Tang et al., 1997; Basu et al., 2015). Secondly, MDx may be used to detect microbes directly from clinical specimens. This reduces the exposure of infection agents and decreases the health risk for the laboratory personnel (Tang et al., 1997). Lastly, the quality and quantity of nucleic acids can be maintained for a prolonged period of time with appropriate storage temperature and specimen preservation (Tang et al., 1997; Leal-Klevezas et al., 2000). This review aims to discuss the advantages and limitations of some MDx techniques for *P. aeruginosa* detection to allow the rapid implementation of infection-control and intervention practices.

## Detection and Identification of *Pseudomonas aeruginosa*

### Polymerase Chain Reaction (PCR)

Polymerase chain reaction (PCR) is one of the notable methods for the detection and identification of *P. aeruginosa* (Deschaght et al., 2011). This method applies primer mediated enzymatic amplification of DNA to synthesize new strand of DNA complementary to the targeted template strand. Different targeted genes have been employed to detect *P. aeruginosa* on clinical samples such as *ecfX*, *oprL*, and *gyrB* due to their high sensitivity and specificity (De Vos et al., 1997; Qin et al., 2003; Anuj et al., 2009). False-positive results (using 16S rRNA and *oprI* genes) and false negative results (using *algD* and *toxA* genes) have also been reported previously (Qin et al., 2003; Cattoir et al., 2010). This may due to the extensive genomic plasticity (Shen et al., 2006) and horizontal gene transfer of this bacterium to other *Enterobacteriaceae* species (Anuj et al., 2009). Furthermore, the comparative-genomics information and inter- or intra-species sequence polymorphisms within the target region are unknown (Qin et al., 2003). To improve aforementioned issues, multiplex-PCR using parallel testing of more than one targeted gene may be used. Multiplex-PCR is able to provide internal controls, lower the reagent costs, preserve precious samples and determine the quality and quantity of template more effectively (Edwards and Gibbs, 1994; Elnifro et al., 2000). The major drawback of this assay is primer designing. The primer–primer competition and relative abundance of target in regard to primer concentration need to be taken into consideration. To date, no standard protocol for the detection of *P. aeruginosa* using multiplex-PCR is available despite of the development and continuous improvement of this approach (De Vos et al., 1997; da Silva Filho et al., 2004; Anuj et al., 2009; Thong et al., 2011; Salman et al., 2013; Aghamollaei et al., 2015).

In recent decades, the advert of the quantitative real-time PCR (qPCR) is gaining popularity for detection of pathogens in clinical microbiology (Klein, 2002; Mackay, 2004; Sails, 2004; Kaltenboeck and Wang, 2005; Valasek and Repa, 2005; Espy et al., 2006; Kubista, 2008; Bustin et al., 2009; Saeed and Ahmad, 2013). This technology only requires less than 5 h, is simple, reproducible, and improved quantitative capacity over conventional PCR (Fournier et al., 2013). Such a powerful tool could be applied for the detection of *P. aeruginosa* directly from sputum samples of CF patients, positive blood cultures, corneal samples, and chronic wounds. To simplify and standardize the experimental design, commercially distributed kits for the detection of *P. aeruginosa* are available in the market. Unfortunately, the instrument for qPCR is expensive for extra light sources and filters for fluorescence detection and may require high cost of maintenance.

### Isothermal Amplification Methods

Current advancement in PCR has led to the development of isothermal amplification methods, including loop-mediated isothermal amplification (LAMP) and polymerase spiral reaction (PSR). The isothermal amplification method requires only basic inexpensive equipment (i.e., standard heat block) with minimal operator training (Diaz and Winchell, 2016) and is capable of providing reliable results within 1 h. This method is useful for clinical screening, especially under lack of resources or for point-of-care testing.

LAMP is a unique nucleic acid amplification technique that amplifies few copies of DNA into billion copies within an hour under isothermal conditions with greater specificity. In LAMP reaction, the gene was amplified when self-elongation of templates from the stem loop structure formed at the 3^′^-terminal plus the binding and elongation of new primers to the loop region (Notomi et al., 2000). In a previous study, the development and validation of LAMP assays for 426 clinical samples (including 252 *P. aeruginosa* and 174 non-*P. aeruginosa* isolates) were performed (Zhao et al., 2011). This study showed the detection limit of extracted DNA was 2.8 ng/μl, while sensitivity of LAMP and PCR assays was found to be 97.6% (246/252 *P. aeruginosa* isolates) and 90.5% (228/252 *P. aeruginosa* isolates), respectively; with a 100% specificity for both assay. Furthermore, LAMP enables direct detection of *P. aeruginosa* from clinical patient plasma within 20 min without the requirement for DNA purification (Yang et al., 2016). The disadvantages of LAMP are proper primer designing required and LAMP multiplexing approach is less developed compared to PCR. On the other hand, PSR is a nucleic acid amplification method based on the utilization of a DNA polymerase with strand displacement activity under isothermal conditions. In China, PSR was developed for rapid detection of *P. aeruginosa* by targeting *toxA* gene within 60 min without an initial denaturation step as required by LAMP. The detection limit of extracted DNA was 2.3 pg/μl and 10-fold more sensitive than conventional PCR, where the reaction proceeds as soon as the temperature reaches 61°C to 65°C (Dong et al., 2015).

## Molecular Typing Methods of *Pseudomonas aeruginosa*

### Pulsed-Field Gel Electrophoresis (PFGE)

Pulse-field gel electrophoresis (PFGE) is for separation of large DNA molecules ranging from 10 kb to 10 Mb on a solid matrix by applying an electric field that periodically changes direction (Kaufmann, 1998). It is a popular method for large-scale epidemiological investigations due to its discriminatory powers (DIs) (Selim et al., 2015; Tang et al., 2017). The DIs expresses the homogeneity of distribution between types and valuable for defining the best typing strategy and interpreting data (Grundmann et al., 1995). The combination of PFGE and restriction endonuclease digestion from genomic DNA isolated from bacterial isolates has proved to be a useful epidemiological tool. For instance, PFGE-SpeI of *P. aeruginosa* was found to have extremely high DIs between 0.98 and 0.998 (Grundmann et al., 1995). Besides, PFGE is a relatively inexpensive approach with excellent typeability, high sensitivity (Abu-Taleb et al., 2013), intra-laboratory reproducibility and easy interpretation (Römling and Tümmler, 2000; Ballarini et al., 2012). PFGE-SpeI approach is the recommended method for *P. aeruginosa* genomes study (Morales et al., 2004; Libisch, 2013). Macrorestriction patterns generated by restriction enzyme SpeI showed 100% reproducibility and the typeability was in between 95 and 100% (Grundmann et al., 1995). One major drawback of PFGE for *P. aeruginosa* typing is the lack of standardized protocols, causing limited intra- and inter-laboratory reproducibility. As improvement, the development of standardized PFGE protocol (culture to gel image) is recommended. Another notable disadvantage of PFGE typing is its labor-intensive method which requires few days to obtain results and involves technical expertise to handle it.

### Multiple Locus Variable-Number Tandem Repeat Analysis (MLVA)

Multiple locus variable-number tandem repeat analysis (MLVA) is a PCR-based method to subtype microbial strains on the analysis of variable copy numbers of tandem repeats (VNTR). MLVA utilizes the naturally occurring variation in the number of tandem repeated DNA sequences found in multiple loci or regions in a bacterial genome detected by PCR using flanking primers (Sabat et al., 2003; de Filippis and McKee, 2012; Sobral et al., 2012). MLVA is more favorable as it appears to contain greater diversity and, hence, greater discriminatory capacity when compared to other type of molecular typing system (Keim et al., 2000). The MLVA scheme for *P. aeruginosa* was first developed by Onteniente et al. (2003) and was subsequently improved by adding new epidemiologically information markers (de Filippis and McKee, 2012). It is a promising tool for molecular surveillance of *P. aeruginosa* in the public health as it is highly reproducible, easy to use and interpret (Onteniente et al., 2003; de Filippis and McKee, 2012; Maâtallah et al., 2013). Besides, MLVA is also a rapid approach with high resolution, thus is beneficial for resolving large and complex outbreak situations. This method is also suitable to apply on commercial kits and large-scale automated platforms (e.g., pipetting robots and automated sequencers) which are already available at the markets (Llanes et al., 2013). Almost 100 isolates could be genotyped in less than 4 days, starting from bacterial colonies using the genotyping kit TYPPSEUDO ceeramTools^®^ (Ceeram, La Chapelle-sur-Erdre, France) and automated capillary-based MLVA system (Sobral et al., 2012). A potential drawback of MLVA method is that inter-laboratory comparison studies cannot be conducted directly. This is mainly due to the fact that the generated amplicons are monitored as banding patterns by conventional electrophoresis on agarose gels, causing difficulty to determine which band in a pattern corresponds to which PCR target. Besides, this approach is also high assay-specific for different organisms and lacks standardization for the majority of published assays (Sabat et al., 2013).

### Multilocus Sequencing Typing (MLST)

Multilocus sequencing typing (MLST) analysis is an electronically portable, universal, and definitive bacterial typing method that focuses solely on conserved housekeeping genes and the combination of each allele (Wendt and Heo, 2016). This is aim to define the sequence type for each isolate and provide information in the relatedness of bacterial isolates at the core genome level. This method has become a very well-known (or popular) tool for molecular evolution studies of pathogens and global epidemiological studies. MLST scheme has been first developed for *P. aeruginosa* by Curran et al. (2004). The study revealed that *P. aeruginosa* is best described as non-clonal but has highly successful epidemic clones or clonal complexes population (Curran et al., 2004). One of the great advantages of MLST is the accessibility of online-based MLST reference databases, allowing this method to gain widespread popularity as an epidemiological tool for bacterial typing. Currently, the MLST databases can be open accessed at http://pubmlst.org/databases and http://www.mlst.net/databases/. The standardization of MLST data allows users to investigate the molecular evolution of pathogens over time in different geographic regions. For outbreaks surveillance and management, MLST being able to rapidly keep track of infectious diseases is of paramount importance (Chui and Li, 2015). MLST is also a technically straightforward typing tool and provides unambiguous data that is highly reproducible between laboratories (Pérez-Losada et al., 2011). Unfortunately, the great disadvantage of MLST is its high cost and insufficiently discerning for routine use in local surveillance and outbreaks. Additionally, the MLST depends on housekeeping genes which are relatively conserved to establish genetic relatedness between isolates as it may lack the discriminatory power to differentiate certain bacteria (Noller et al., 2003; Pérez-Losada et al., 2011).

### DiversiLab Repetitive-Sequence-Based PCR (DL rep-PCR)

DiversiLab (DL), an automated repetitive-sequence-based PCR (rep-PCR) bacterial typing system (bioMérieux), comes with a high level of standardization, particularly at the electrophoresis step by using the Bioanalyzer (Agilent Technologies, Inc., Santa Clara, CA, United States) (Healy et al., 2005; Deplano et al., 2011). In comparison to traditional gel electrophoresis, DL system makes use of microfluidic capillary electrophoresis to overcome the low reproducibility of the previous rep-PCR approaches (Sabat et al., 2013). Besides, DL system also provides a user-friendly internet-based computer-assisted data analysis (Fluit et al., 2010; Brossier et al., 2015). The reliability of the data obtained from the system is species dependent. Hence, a validation for each bacterial species is necessary prior to its use in a routine clinical laboratory (Harrington et al., 2007; Doleans-Jordheim et al., 2009). Another major drawback of DL system for *P. aeruginosa* is the lack of a suitable cutoff values from the manufacturer. The classification of the closely related DL rep-PCR profiles as identical genotypes may be influenced by the number of different fragments obtained due to deletions, insertions, inversion of DNA, or mutation of the restriction. For outbreaks or routine clinical laboratory bacterial typing practices, the high cost of reagents and kits as well as the necessity to use different fingerprint kits for each bacterial species are among the disadvantages of DL system which need to be overcome. Moreover, the initial instrument installation and maintenance costs also need to be taken into consideration.

## The New Era of Molecular Diagnostics: Next Generation Sequencing (Ngs) Technology

Over the years, next generation sequencing (NGS) technology has gradually been replacing the first generation sequencing, the Sanger sequencing (Sboner et al., 2011; Muir et al., 2016). The NGS technology is evolving into a molecular microscope, which manages to provide a broad investigations of the bacterial genomes (e.g., transcription, translation, replication, methylation, and nuclear DNA folding) and are readily applied in clinical microbiology to replace conventional characterization studies of pathogens (Didelot et al., 2012; Gargis et al., 2012, 2016; Behjati and Tarpey, 2013; Deurenberg et al., 2017; Tagini and Greub, 2017).

Since first introduced to the market in 2005, NGS is widely accepted, allowing decentralized laboratories to conduct their own internal genome sequencing projects at a lower cost (Quail et al., 2012; Liao et al., 2015). Besides, NGS also relatively requires less amount of DNA to produce accurate and reliable data. This technology has greater advantages in producing high quality, robustness and lower noise background sequence data. Unfortunately, a successful NGS project requires expertise to perform the wet lab, analyze, and interpret the data (Buermans and den Dunnen, 2014; Ari and Arikan, 2016). In addition, users need to face technical challenges where the current available computational infrastructures and software need to be upgraded in order to store and analyze large bioinformatics datasets. This will be a constant challenge for users as sequencing technology continues to evolve and develop new computational strategies (Tang et al., 2017).

### NGS Applications of Clinical *Pseudomonas aeruginosa*

**FIGURE 1:**
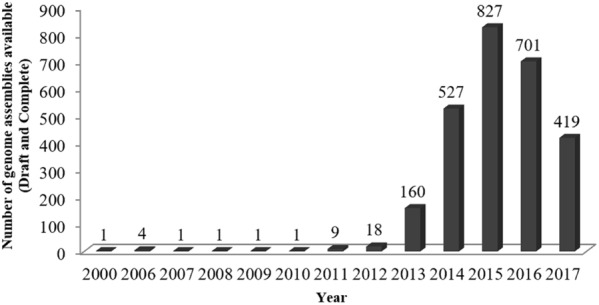
Number of *Pseudomonas aeruginosa* genomes available in NCBI database from year 2000 to 2017.

The applications of NGS for clinical *P. aeruginosa* are wide-ranging and include 16S rRNA gene sequence, whole genome sequencing (WGS), transcriptome profiling, exome sequencing, virtual resistance testing, and public health surveillance. This eventually increases the number of publicly available *P. aeruginosa* genomes (**Figure 1**). Hitherto, there are a total of 2678 assembled genomes deposited in NCBI database^1^ and among them 106 are completed genomes (retrieved in January, 2018). Knowledge on the bacterial genomes helps to harbor information about the genomes evolution, niche adaptation, infectious potential (Bowlin et al., 2014; Dubern et al., 2015; Bartell et al., 2017) and the molecular basis of antimicrobial resistance (Ramanathan et al., 2017; Sherrard et al., 2017). Throughout the years, this technology has facilitated to reveal the pathogenesis of this bacterium. For examples, it is opined that NGS can be used not only to study the bacteria genome, but it can also be used to study the antimicrobial resistance (Chan, 2016). Also, the studies by Kos et al. (2015) and Ramanathan et al. (2017) demonstrated the utility of NGS to define relevant resistance elements as well as highlight the diversity of resistance determinants within *P. aeruginosa*. This information is especially valuable for diagnostics, therapeutics, and preventions of *P. aeruginosa* infections.

Studies on the bacterial genomes also can provide information about the relationship of different pathogens which can be used as source tracking during infection outbreaks. In recent years, NGS coupled with WGS can be used for source tracking of *P. aeruginosa* in a hospital setting, and that acquisitions can be traced to a specific source within a hospital ward (Quick et al., 2014). Besides, WGS could also be applied for epidemiological investigation of *P. aeruginosa* in intensive care units (ICU) (Blanc et al., 2016). Recent years, NGS has been adapted for 16S rRNA gene based metagenomics study to rapidly catalog the bacterial species in mixed clinical specimens, without need for prior culture (Lim et al., 2014). Cummings et al. (2016) demonstrated that NGS 16S rRNA gene sequencing has generated a reproducible, analytically sensitive, and accurate assessment of the identity and relative abundance of organisms present in polymicrobial samples, outperforming standard culture.

## Conclusion

Over the last decades, several molecular methods have been developed for genotypically detecting and identifying pathogens in clinical diagnostic laboratories. All the methods discussed here have both advantages and drawbacks or rather limitations(**Table 1**). Another trend for microbial identification which relies on microbial physiological or biochemical characteristics including antibiotic-resistance has been fast developed from well-established analytical profile index (API) to mass spectrometry (MS)-based methodologies. Nevertheless, the enrichment of bacterial cells and metabolites for detection and lack of biomarker database are still the major challenges (Cheng et al., 2016). Coupling with genetic and non-genetic methods have also been developed such as PCR-MS for determining nucleotide compositions of strain-specific PCR products (Ecker et al., 2005). Further studies of both genotypic and phenotypic methodologies will certainly facilitate the development of perfect diagnostic, which is rapid, specific, sensitive, easy to perform and interpret, cost-effective, and high-throughput.

**Table 1:** Comparison table of selected molecular techniques.

Molecular techniques	Advantages	Limitations	Reference
PCR	∙ High sensitivity ∙ High specificity	∙ False-positive results ∙ Negative results	Qin et al., 2003; Cattoir et al., 2010
Multiplex PCR	∙ Provide internal controls ∙ Low reagent costs ∙ Able to preserve precious samples ∙ Able to determine the quality and quantity of template more effectively	∙ Primer designing ∙ No standard protocol	De Vos et al., 1997; da Silva Filho et al., 2004; Anuj et al., 2009; Thong et al., 2011; Salman et al., 2013; Aghamollaei et al., 2015
qPCR	∙ Reproducible methods (less than 5 h) ∙ Direct detection from sputum samples ∙ Availability of commercial kits in the market	∙ Expensive instrument ∙ High cost of maintenance	Deschaght et al., 2009; Cattoir et al., 2010; Clifford et al., 2012; Carlesse et al., 2016
LAMP	∙ Low detection limit with high sensitivity ∙ Rapid detection (∼20 min) without DNA purification ∙ Only required basic inexpensive equipment with minimal operator training	∙ Primer designing ∙ Less develop multiplexing approach	Zhao et al., 2011
PSR	∙ Low detection limit with high sensitivity ∙ Rapid detection (∼60 min) without an initial denaturation ∙ Only required basic inexpensive equipment with minimal operator training	∙ Still in the progress on method development	Dong et al., 2015
PFGE	∙ Inexpensive ∙ Excellent typeability ∙ High sensitivity ∙ Easy interpretation	∙ Lack of standardized protocols ∙ Limited reproducibility ∙ Labor-intensive method ∙ Technical expertise required	Grundmann et al., 1995; Morales et al., 2004; Libisch, 2013
MLVA	∙ Highly reproducible and easy interpretation ∙ Rapid approach with high resolution ∙ Suitable for large-scale automated platforms	∙ Assay-specific for different organisms ∙ Lacks standardization of assay	Onteniente et al., 2003; Sobral et al., 2012; Maâtallah et al., 2013
MLST	∙ Accessibility of online-based MLST reference databases ∙ Standardization of MLST data ∙ Highly reproducible	∙ High cost ∙ Insufficiently discerning for routine use in local surveillance and outbreaks ∙ Lack the discriminatory power to differentiate certain bacteria	Curran et al., 2004
DL rep-PCR	∙ Standardization of assay ∙ Improved reproducibility ∙ User-friendly internet-based computer-assisted data analysis	∙ Validation for each bacterial species is necessary ∙ Lack of a suitable cutoff values from the manufacturer ∙ High cost of reagents and kits ∙ Necessity to use different fingerprint kits for each bacterial species ∙ High instrument installation and maintenance costs	Fluit et al., 2010; Deplano et al., 2011; Brossier et al., 2015
NGS	∙ Requires less amount of DNA ∙ High quality, robustness and lower noise background sequence data ∙ Reproducible ∙ Analytically sensitive, and accurate assessment of the identity and relative abundance of organisms present in polymicrobial samples	∙ Technical expertise required to perform the wet lab, analyze, and interpret the data ∙ Computational infrastructures and software need to be upgraded in order to store and analyze large bioinformatics datasets	Quick et al., 2014; Blanc et al., 2016

PCR, polymerase chain reaction; qPCR, quantitative real-time PCR, LAMP, loop-mediated isothermal amplification; PSR, polymerase spiral reaction; PFGE, pulsed-field gel electrophoresis; MLVA, multiple locus variable-number tandem repeat analysis; MLST, multilocus sequencing typing; DL rep-PCR, DiversiLab repetitive-sequence-based PCR; NGS, next generation sequencing.

## Author Contributions

J-WC and YYL contributed to data collection, analysis, and draft the manuscript. TK, K-GC, and C-YC revised the manuscript. All authors read and approved the final version of the manuscript.

## Conflict of Interest Statement

The authors declare that the research was conducted in the absence of any commercial or financial relationships that could be construed as a potential conflict of interest.

## Footnotes

1 http://www.ncbi.nlm.nih.gov
